# Risk-Based Consumption of Dioxin-Contaminated Farmed Salmon

**DOI:** 10.1289/ehp.113-a655

**Published:** 2005-10

**Authors:** John P. Middaugh, Scott M. Arnold, Lori A. Verbrugge

**Affiliations:** Alaska Division of Public Health, Anchorage, Alaska, E-mail: john_middaugh@health.state.ak.us

In their article, “Risk-Based Consumption Advice for Farmed Atlantic and Wild Pacific Salmon Contaminated with Dioxins and Dioxin-like Compounds,” Foran et al. (2005) present recommendations for consumption of salmon containing dioxin-like compounds (DLCs) based on three risk assessment approaches.

Relying strictly on risk assessment to develop fish consumption advice has many shortcomings and may actually do more harm than good (Arnold et al. 2005; Egeland and Middaugh 1997). Risk assessment is only part of the risk management process when developing fish consumption advice [U.S. Environmental Protection Agency (EPA) 1996]. Other factors need to be considered when developing fish consumption advice, such as the nutritional and health benefits of consuming fish and the cultural, societal, and economic impacts of reduced fish consumption (U.S. EPA 1996). Ignoring these factors may place an undue burden on a local population by removing a relatively inexpensive protein source that would likely be replaced by a less healthy substitute (Arnold et al. 2005; Egeland and Middaugh 1997). Decisions to limit fish consumption should only be made at the local level because local public health officials are most aware of the local aforementioned impacts and the actual concentration of contaminants in locally caught fish [Arctic Monitoring and Assessment Programme (AMAP) 2002; Arnold et al. 2005; Hites et al. 2004].

In addition to measuring contaminant concentrations in fish, human biomonitoring is a very useful tool to measure actual levels of contaminant exposure in targeted “at-risk” populations rather than relying solely on calculated estimates of exposure. Biomonitoring should ideally be performed in any identified at-risk population to verify that a problem actually exists before advising people to reduce fish consumption. This is especially true in populations that rely on locally caught fish as their primary protein source and that have few inexpensive healthy alternatives. Fortunately, recent evidence suggests that the average concentration of DLCs in the general U.S. population is declining (U.S. EPA 2000).

Two aspects of this study’s (Foran et al. 2005) methodology are problematic because they lead to inappropriately conservative estimates of health risk. First, the majority of people in the United States do not eat salmon skin, and as the authors noted, cooking has been shown to reduce DLCs in fish tissue. Because DLCs partition to fatty tissues including skin, measuring DLCs in raw fish with the skin on will overestimate the amount of exposure to DLCs and overestimate the consumption risks. Second, when assessing health risks posed by salmon consumption, Foran et al. (2005) estimated the number of meals of salmon per month that would limit dioxin intake to 20% above the U.S. average “background” adult intake level of 65 pg toxic equivalents (TEQ)/day. This approach ignores the fact that the background rate incorporates both freshwater and saltwater fish consumption (U.S. EPA 2000). In effect, Foran et al. (2005) double counted dioxin exposure through fish consumption. They also ignored the fact that a person who chooses not to consume a salmon meal would likely substitute another protein source that also contains trace quantities of DLCs.
